# Psychological Interventions for Cannabis Use among Adolescents and Young Adults: A Systematic Review

**DOI:** 10.3390/ijerph20146346

**Published:** 2023-07-12

**Authors:** Yara Bou Nassif, Hassan Rahioui, Isabelle Varescon

**Affiliations:** 1 Laboratoire de Psychopathologie et Processus de Santé, Université Paris Cité, 92100 Boulogne Billancourt, France; isabelle.varescon@u-paris.fr; 2 Consultations en Addictologie pour Adolescent, Centre des Troubles de Neuro-Développement chez l’Adulte, Groupe Hospitalier Universitaire, Site Sainte-Anne, 75014 Paris, France; h.rahioui@ghu-paris.fr

**Keywords:** cannabis, adolescence, young adults, psychological interventions, systematic review, randomized controlled trial

## Abstract

Regular cannabis use during adolescence can lead to cognitive, psychological, and social consequences, causing significant distress. Although psychological interventions are the mainstay type of treatment for cannabis use disorder, the results remain mixed among youths. The objective of this review is twofold: to identify the existing psychological interventions for cannabis use among youths, and to assess the evidence regarding the effectiveness of those interventions. Randomized controlled trials focused exclusively on cannabis use among adolescents and young adults were included. Three databases—Embase, PsycInfo, and PubMed—were searched to identify relevant peer-reviewed manuscripts published before February 2022 in English and French. The risk of bias was assessed using the Cochrane Collaboration’s tool. Twenty-five randomized controlled trials were included. Fourteen studies reported a significant outcome related to cannabis use. These were mainly non-intensive, online interventions that aimed to improve the patients’ relationships and emotion regulation. This review highlights the need to conduct additional randomized control trials that target cannabis use disorder specifically among adolescents. These randomized control trials should also aim to reduce the risk of bias related to psychiatric comorbidities as well as detection and attrition problems.

## 1. Introduction

Cannabis is the most broadly used psychoactive substance in the world after tobacco and alcohol, particularly among adolescents and young adults [1]. Cannabis use disorder (CUD) is defined by the Diagnostic and Statistical Manual of Mental Disorders, 5th Edition (DSM-5), as a problematic pattern of cannabis use (CU) that leads to significant impairment or distress occurring within a 12 month period. The diagnostic criteria include patterns such as exceeding the intended CU, unsuccessful attempts to curtail use, devoting substantial time to obtaining or using cannabis, experiencing intense cravings, impaired fulfillment of obligations, encountering social or interpersonal difficulties, and persistent use despite negative consequences. The severity of the disorder is graded based on the number of met criteria, ranging from mild to moderate to severe [2].

In 2020, in the United States, 10.1% of adolescents aged 12–17 and 34.5% of young adults aged 18–25 reported CU within the previous year. Moreover, these age groups reported the highest percentage of CUD during that period [1]. In Europe, 15.5% of young adults between 15 and 34 reported CU in the last year [3]. Adolescence is a critical period for brain development. Neuromaturation, consisting of synaptic overproduction, pruning, and myelination, facilitates connections between different brain areas, enabling the development of cognitive abilities such as decision making, impulse control, and emotion regulation [4]. As the adolescent brain continues to mature until the age of 25, it is highly vulnerable to external influences, especially psychoactive substances, which can disrupt its development. One significant concern is the impact of substances such as cannabis that target the endocannabinoid system, a crucial player in the maturation of adolescent neurons [4,5,6,7]. The endocannabinoid system serves as a regulatory and homeostatic mechanism that undergoes developmental changes throughout adolescence. Consequently, it becomes more susceptible to the effects of cannabis exposure during this critical period.

While the evidence regarding the causality between adolescent cannabis use and cognitive, psychiatric, and neurobiological differences is still being examined, longitudinal studies have revealed that higher levels of cannabis use during adolescence, characterized by increased frequency and duration, are associated with a higher prevalence of psychiatric disorders and cognitive impairments [4,6,7].

Two recent papers on the IMAGEN study conducted across eight European countries and involving 799 adolescents with an average age of 14 years investigated the effects of cannabis use on brain development [8,9]. In a five-year-long follow-up, the authors discovered that cannabis use was associated with the accelerated thinning of the cortex, particularly in regions linked to the prefrontal cortex. Furthermore, the pattern of thinning observed in these regions was also related to the activity of cannabinoid receptors in the brain.

CU during this developmental stage has been consistently associated with brain alterations, including structural and functional changes [4,6]. These alterations, in turn, contribute to long-term cognitive and psychological risks that can lead to clinically significant distress. Recent meta-analyses demonstrated that the frequency and potency of CU during this period are associated with an increased risk of developing various psychiatric disorders, such as substance use disorders, anxiety, depression, suicidality, and psychosis [5,7,10].

Regular CU during adolescence and young adulthood can thus result in notable cognitive, psychological, academic, and social consequences. Given the pivotal role of these formative years in shaping individuals’ future trajectories, it is imperative to evaluate the effectiveness of targeted interventions aimed at reducing cannabis use within this population.

However, adolescents and young adults appear to be more reluctant to seek help and are more challenging to engage in therapy for substance use disorder [11]. Moreover, interventions for CU among youths have yielded mixed results. “The Cannabis Youth Treatment” was the first study to examine the efficacy of five interventions among 600 adolescents with CUD in two randomized controlled trials (RCT). All five interventions showed significant results in reducing CU one year after randomization, without differences between them [12].

More recently, Halladay et al. [13] demonstrated the efficacy of interventions based on one or two sessions in reducing CUD symptoms and enhancing the likelihood of abstinence among youths who practice infrequent cannabis use. This review included observational studies, quasi-randomized trials, and RCT, and the baseline CU was not a prerequisite. Conversely, Li et al. [14] did not find significant results for brief interventions on CU in postsecondary students, even in the short term. However, in this review, only five RCTs were included based on secondary prevention for substance use. Similarly, Beneria et al. [15] concluded that online interventions addressing substance use in general were not effective in reducing CU among youths with a mean age of 15–30 years. In contrast, Olmos et al. [16] found significant results for internet- or computer-based interventions targeting cannabis and other substances, but this review included only nine RCTs on both adolescents and adults. Likewise, Boumparis et al. [17] found significant results for internet- or computer-based prevention and intervention programs on CU among adolescents and adults with all types of substance use. However, the results at the 12 month follow-up were only maintained for prevention programs.

Recent reviews have only covered digital brief psychological interventions for youths who did not meet the criteria for CUD or who were not identified as engaging in regular CU. They also included interventions combining all substance use and not targeting cannabis exclusively. Therefore, the objectives of this study are:

1. To systematically review all the existing psychological interventions that target CU specifically among youths;
2. To describe the different criteria, methodologies, and frameworks used in these interventions;
3. To assess the effectiveness of different techniques employed in interventions and guide future evaluations during this critical time of development.

## 2. Materials and Methods

### 2.1. Eligibility Criteria

We included only the RCTs evaluating a psychological intervention for CUD in comparison to that for a control group, including active comparators or no interventions. We included studies on adolescents and young adults up to 25 years old, reporting CU as a primary outcome, regardless of the CU level, who received intervention in an outpatient setting. We excluded studies with primary prevention and pharmacological interventions as a control group and those on acute psychiatric comorbidities, such as major depressive disorder or psychotic disorders and substance use disorders other than cannabis and tobacco.

The interventions were delivered in an individual or group format, online, over the phone, computer, or in person.

Studies were included that assessed efficacy as a primary outcome in terms of CU frequency and quantity via self-reporting or biological analysis, CUD severity, and cannabis-related problems using standardized questionnaires or clinical assessments. Secondary outcomes included frequency and quantity of other substance use, craving, psychopathology or psychosocial functioning, attendance, and retention and dropout rates.

### 2.2. Searching Strategy

This review followed the PRISMA guidelines for systematic reviews. Before starting, we registered the protocol on PROSPERO (registration number: CRD42022302285).

Two researchers conducted the inclusion process and searched three databases (Embase, PsycInfo, and PubMed) with the following keywords: (intervention OR psychotherap* OR therap* OR program OR treatment OR counsel*) AND (cannabis OR mari?uana OR hashish) AND (young* OR adolescen* OR juvenile OR student* OR teen* OR youth OR college OR school* OR minor* OR “emerging adult*” OR “early adult*” OR junior* OR pubescen*) AND (random*), in order to assess English and French, peer-reviewed manuscripts published before February 2022. In addition, we conducted a manual search for “grey” literature databases (ResearchGate and Google Scholar) to supplement our search. The findings were then exported to Zotero. Data extraction was recorded by one author on an Excel spreadsheet and included publication details, sample characteristics, intervention characteristics measurement outcomes, follow-up times, and main outcomes. A second author reviewed the extracted data, and disagreements were resolved via a discussion.

The risk of bias (ROB) was assessed using the Cochrane Collaboration’s tool [18], which examines seven domains: random sequence generation, allocation concealment, the blinding of participants and personnel, the blinding of outcome assessment, incomplete outcome data, selective reporting, and other biases. This tool determines an overall ROB grade of high, low, or unclear. The authors assessed the ROB of all the articles independently. Disagreements were resolved through a discussion.

## 3. Results

### 3.1. Characteristics of Included Studies

**Figure 1:**
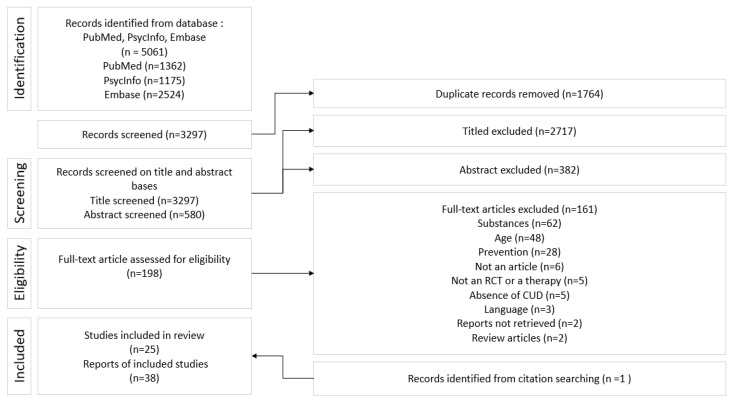
Flow diagram of the study selection.

A total of 5061 articles were identified. After removing 1764 duplicates, we screened 3297 records for titles and abstracts, sorting them according to the inclusion criteria (see Figure 1). 25 RCTs (38 reports) met the inclusion criteria and involved 4077 participants. 22 RCTs took place in the United States, one took place in Australia [19], one took place in the Netherlands [20], and one took place in the United Kingdom [21].

Based on the extracted data, the results were synthesized according to intervention and participant characteristics, ROB, and intervention effects. Of the 25 RCTs included, 16 interventions were conducted in person, eight were conducted online, and one study compared the same intervention delivered via computer or therapist to a control group (see Table 1). In addition, nine studies used an active treatment as a control condition.

The psychosocial treatments for CUD include motivational interviewing/enhancement therapy (MI/MET), cognitive and behavioral therapy (CBT), parent training, working memory training, and contingency management (CM) techniques.

MI/MET emphasizes building motivation and self-efficacy in a supportive environment. CBT focuses on identifying and managing patterns, triggers, and thoughts associated with CU, while teaching coping skills and promoting healthier behaviors. Parent training enhances communication and helps parents manage adolescents’ behaviors. Psychoeducation provides information about cannabis and its effects, working memory training targets cognitive deficits associated with CUD. CM utilizes positive reinforcement and incentives to encourage abstinence from CU.

### 3.2. Types of Interventions

#### 3.2.1. In-Person Interventions

In the face-to-face interventions, ten were based on MI, five were based on MI and CBT, and two were based on cognitive training. Four of these interventions added a CM component for abstinence or attendance [22,23,24,25], and three studies added a family component [23,24,26].

All of the in-person interventions reported some part of the practitioner’s practice, but a majority did not specify the degree, certification, or training involved, such as a bachelor-, master-, and/or doctoral-level counselor or clinician [22,23,24,26,27,28,29,30]; a doctoral-level, graduate student, or doctoral-level professional [31]; a prevention worker [15]; peer educator [32]; counselor or therapist [19,33]; research therapist [34]; or an academic researcher, psychology graduate, or college-based practitioner [16]. However, in all of these studies except one [28], the adherence to treatments was verified via audio or videotapes.

Across the in-person interventions, the treatment duration varied from one session [16,26,27,29] to two sessions [14,15,21,22,23,24] to 14 weeks [18,19].

#### 3.2.2. Interventions with Parents

Among the interventions with parents, one study examined the efficacy of adding a session with the parents based on adolescent risk behaviors (family checkup) in comparison to that of psychoeducation [21]. During the family checkup, the focus was on building positive relationships, limit setting, and problem solving, whereas in psychoeducation, the focus was on education about cannabis and its effects [21]. The other authors assessed 14 sessions with the parents based on family management and compared them to the psychoeducation ones. In the family management sessions, the objectives were to identify adolescent risk behaviors and to develop incentives and consequences in order to establish a substance-monitoring contract. For the parent psychoeducation study, the focus was on substances and their consequences and on parenting strategies [18,19].

#### 3.2.3. Online Interventions

All the online interventions used an MI approach, five of which were based on personalized feedback interventions (PFI) [35,36,37,38,39], and two were based on text messages [40,41].

The online interventions examined the effectiveness of integrating MI and CBT with the presence of an e-coach [42], psychoeducation [31], peer relationships [35,36], or a negative affect [30].

Overall, one study evaluated the effect of combining in-person intervention with a mobile application based on self-monitoring and feedback messages [22], while another study compared the same intervention delivered via computer or a therapist [29].

#### 3.2.4. Control Groups

Regarding the control groups, four online interventions [35,36,37,40], and two in-person interventions used an assessment-only group [31,32], whereas one online [41] and two in-person interventions used a waiting list as a control group [19,28].

Among the active control group, the authors evaluated personalized normative feedback only [39], healthy stress management [38], CM [25], educational feedback [29], MET [21,22,25], MET and CBT with or without CM [17,18,19,28], drug counseling with or without CM [16,17], cognitive training [43], brochure or information session [15,29], and online content unrelated to substance use and mental health [37]. Two of these interventions added a parent psychoeducational variable [18,21].

### 3.3. Participants

The participants were high school or university students, or attended an outpatient program (see Table 1). The age groups varied across studies: 18–25 (*n* = 10), 15–24 (*n* = 1), from 16 or 17 to 19 (*n* = 2), from 14 to 19 (*n* = 4) or 21 (*n* = 3), 12–26 (*n* = 1), and from 12 or 13 to 18 (*n* = 4).

The inclusion criteria related to CU were also different across articles. Nineteen studies used a cutoff score, and only six assessed the diagnosis of CUD. The cutoff was heterogeneous, ranging from use during the past year to at least three times per week. In addition, some studies did not specify a period for CU as an inclusion criterion, such as weekly CU [22].

Among the 25 included studies, nine did not report any exclusion criteria. 16 mentioned a substance use disorder other than nicotine or cannabis as an exclusion criterion. The substance use disorder criteria varied across studies, with some based on established diagnostic instruments such as the Vermont Structured Diagnostic Interview (*n* = 4), the DSM (*n* = 1), or the drug abuse screening test-10 (*n* = 1). Other studies referred to criteria such as previous treatment for substance use (*n* = 4), the current need for inpatient treatment (*n* = 3), and/or the quantity or frequency of other substance use (*n* = 3).

Additionally, 11 studies identified psychiatric disorders as exclusion criteria. Among those studies, three specified the disorder assessed and four indicated the assessment tool, such as the DSM-IV [38] or DSM-5 [20], the Vermont Structured Diagnostic Interview [18], and the Adolescent Diagnostic Interview. Moreover, only six studies assessed substance use other than cannabis, and only nine added an objective assessment such as urine drug screening.

Note that in our review, a substance use disorder related to something other than nicotine or cannabis, as well as acute psychiatric conditions, were exclusion criteria. Therefore, we could not include “The Cannabis Youth Treatment” because many adolescents in that study presented other substance use disorders and severe psychiatric disorders [7].

### 3.4. Risk of Bias

Concerning selection bias, 8% of the included studies had a low ROB in the random sequence generation, and 72% did not specify allocation concealment, resulting in an unclear ROB. No study presented a high ROB in this domain. For performance bias, 92% presented a high ROB, as the blinding of participants and personnel was not possible due to the nature of the interventions. Two RCTs had a low ROB, since the intervention and control groups were similar and computerized [34,38]. Therefore, only four studies had a low ROB in the blinding of the subjective outcome assessment, given that it was a hetero evaluation, while the others were self-assessments and had a high ROB. However, nine studies added a urine drug screen test to assess the CU outcomes and had a low ROB. For attrition rates, 56% studies used the appropriate analysis of missing data, while eight RCTs performed simple imputations (e.g., last observation carried forward), and three had a high risk, as they did not report attrition in their analysis. Overall, attrition rates varied from 1% [31] to 57% [19,38]. 11 studies presented an attrition rate greater than 20%, which is considered to lead to a high risk of bias [39].

**Figure 2:**
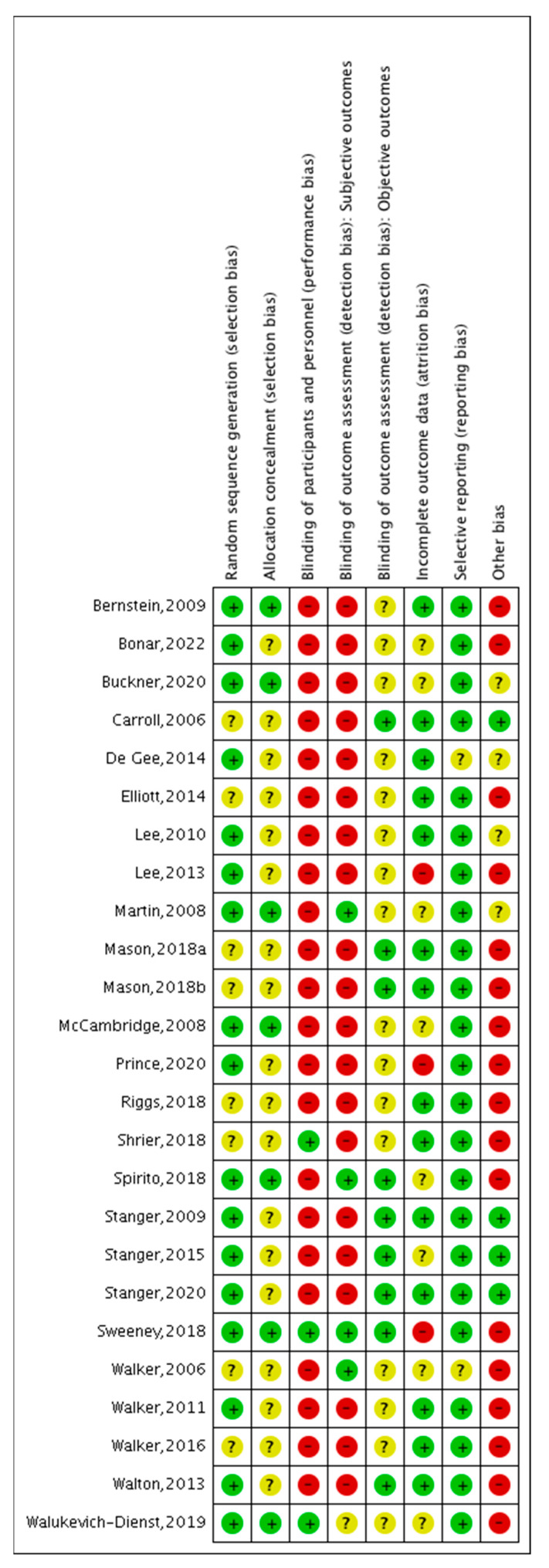
Risk of bias assessment of the included studies [19,20,21,22,23,24,25,26,27,28,29,30,31,32,33,34,35,36,37,38,39,40,41,42,43].

Of the included studies, 92% presented a low ROB concerning a reporting bias. For the other biases, 80% of the studies presented a high ROB, given that they did not measure other confounding variables, such as a history of CUD, psychiatric comorbidities, or access to other treatments during the study period (see Figure 2).

### 3.5. Effects of Interventions

To provide a clear framework for evaluating the effects of the interventions, we categorized them into two main types: in-person and online interventions. Within the in-person interventions, we further classified them based on the approach used, including interventions based on MI/ MET, interventions combining MET and CBT, interventions involving parents, and interventions based on working memory.

#### 3.5.1. In-Person Interventions

##### Interventions Based on MET

Regarding the in-person interventions, Lee et al. [31] reported that the participants receiving a single MI session reduced their amount of CU at the three-month follow-up compared to the amount of CU of the assessment-only control group (*p* < 0.05), but this difference was not maintained at the six-month follow-up. McCambridge et al. [21] also did not find any differences between a single MI session and the drug information and advice-giving session at the three- and six-month follow-ups.

Conversely, another study found more significant results from a 30 min session based on MI delivered by a peer educator compared to those of a control group receiving brief written information [32]. Among the participants who had used cannabis in the past 30 days, those in the intervention group reported significantly more days of abstinence compared to the number of those of the control group (*p* < 0.027) at the 12 month follow-up. They also reported a significantly greater reduction in CU from the baseline to the 12 month follow-up (*p* = 0.024).

Walker et al. [28,29] assessed the efficacy of two 30- to 60 min MET sessions. At the three-month follow-up, they did not find any differences between MET and the delay control group in terms of the frequency or quantity of the ingestion of cannabis and other substances, or CUD symptoms [28]. In addition, Walker et al. [29] found that the participants in the MET and educational feedback control groups reported significantly fewer days of CU and a lower number of CUD symptoms and CU problems than those in the delay control group did at the three-month follow-up, with no difference between groups [29]. However, at the 12 month follow-up, the authors found no differences between the three groups neither in CU outcomes nor in CBT session attendance. After controlling for baseline CU, the CBT sessions were associated with lower amount of CU at the 3 and 12-month follow-ups.

In a more recent study, Walker et al. [30] compared an intervention of two initial MET sessions coupled with three post-intervention MET sessions to a control group of two initial MET sessions. They proposed optional sessions of individual CBT to both groups after completing the intervention. At the six-month follow-up, participants in the intervention group reported fewer days of CU (*p* = 0.01) and fewer CUD symptoms (*p* = 0.01). No other differences were observed at the follow-up assessment. In line with their previous study, there was no difference between groups concerning CBT attendance, but attendance was correlated to a decrease in CU frequency and consequences.

Additionally, Shrier et al. [27] compared three groups, in which all the participants received two sessions of MET. The participants in the “MOMENT” group had access to mobile self-monitoring and feedback messages; those in the “No Message” group only had access to mobile self-monitoring; those in the “MET Only” group did not have access to neither mobile self-monitoring nor feedback messages. At the three-month follow-up, the authors found no differences between the three groups. Concerning momentary assessments, craving was less important in the “MOMENT” group than it was the “MET Only” group (*p* = 0.006), and CU following a trigger was less important in the “MOMENT” (*p* = 0.01) and “No Messages” groups (*p* = 0.02) than it was in the “MET Only” group.

##### Interventions Based on MET and CBT

Martin and Copeland [19] assessed the intervention “Adolescent Cannabis Check-Up” (ACCU), comprising two sessions on MI and CBT compared to a delayed treatment control. The participants in the ACCU group decreased their CU frequency (*p* = 0.032) and quantity (*p* = 0.021) in the previous 90 days and reduced their number of CUD symptoms (*p* = 0.04) compared to those of the control group. However, the participants in the ACCU group reported more CU at the baseline than the control group did.

Similarly, De Gee et al. [20] compared two sessions inspired by the ACCU to an information session. At the three-month follow-up, the authors did not find differences between the groups in any variable. The only difference concerned heavier CU, such as those in the intervention group, who significantly reduced CU quantity more than the control group did. Additionally, a recent study compared four sessions of MET and CBT, with and without physical exercises [33]. At the six-month follow-up, the participants in the exercise group significantly reduced their CU quantity more than those in the control group did. The use of a protective behavioral strategy also predicted a lower quantity of CU.

Carroll et al. [22] compared four groups of young adults referred by the criminal justice system in MET and CBT both with and without CM and drug counseling (DC) with and without CM over eight weeks. CM was based on attendance or on a negative urine drug screen result. The authors found that MET, CBT, and CM, as well as CM, were significantly more effective for treatment retention. During the treatment period, both the CM groups completed significantly more sessions, continuous days of abstinence (*p* = 0.04), had a negative urine drug screen result (*p* = 0.01), and more consecutive negative urine tests (*p* = 0.01) than the groups without CM did. Similarly, the participants in the MET, CBT, and CM groups had significantly more consecutive negative urine drug screen results (*p* < 0.05) and a smaller number of positive urine drug screen results (*p* < 0.05) than the MET and CBT group or the DC group did. However, at the 6 month follow-up, the only difference observed was that participants in the MET and CBT groups more significantly reduced their CU frequency than those in the DC group did.

##### Interventions with Parents

Stanger et al. [23] compared an intervention of MET, CBT, and CM based on abstinence and family management to a control group of MET, CBT, and CM based on attendance and parent psychoeducation. During the treatment, the participants in the intervention group reported significantly more mean weeks of continuous abstinence (*p* = 0.04), as well as 10 or more weeks of continuous abstinence (*p* = 0.006) based on urine drug screening. There were no differences in CU between groups at the follow-up assessment based on the urine drug screen or self-reporting results. Furthermore, Stanger et al. [24] compared an intervention based on MET, CBT, CM, and parent training to an intervention based on MET and CBT with and without CM. During the treatment, the participants in both CM groups (with or without parent training) completed more continuous abstinence based on urine drug screening than those in the MET/CBT group did. All three groups did not differ regarding CU frequency. At the end of treatment, the participants in the MET, CBT plus CM group reported higher rates of abstinence than those in the MET/CBT group did (*p* ≤ 0.01); this difference was not significant at any follow-up. Parent training did not have an impact on CU, even though parents could continue to come to the clinic for 12 more weeks to continue parental monitoring.

##### Interventions Based on Working Memory

Two other studies assessed an intervention based on working memory. Stanger et al. [25] randomized the participants in an intervention based on CM and cognitive training or based on CM only. After four weeks of treatment, non-abstinent participants were again randomized to their initial group, with or without enhanced CM. At the end of the treatment, the groups did not differ in terms of CU or on working memory. The authors did not find any cognitive training benefits or enhanced CM regarding CU or cognitive functions. Sweeney et al. [43] compared cognitive training involving an adaptive procedure to a static procedure. The only difference concerned emotion regulation, a variable of psychosocial functioning, with a significant difference in favor of the control group (*p* < 0.001). There were no differences between groups in CU frequency and quantity, in other psychosocial functioning, or other substance use.

#### 3.5.2. Online Interventions

Five online interventions were based on PFI and specifically targeted undergraduate students (see Table 2). Among them, Elliott et al. [36] found that the participants in the educational program did not see any more reductions in the frequency, consequences, or symptoms of CUD compared to those of the assessment control group. However, women in the intervention group reported fewer CUD symptoms than those in the control group did, whereas men reported more CUD symptoms than those in the control group did. Similarly, Lee et al. [37] evaluated a comparable online PFI and reported no difference between the groups in terms of CU frequency or in CU consequences at the three- and six-month follow-ups. Only those with a family history of substance use in the intervention group decreased their CU more than those without any family history of substance abuse did (*p* = 0.01).

A recent study comparing an online PFI focused on the negative effects felt by an assessment-only group and reported no differences between groups in terms of CU, social anxiety, or effects [35]. Yet, the level of social anxiety moderated the relationship between intervention and CU. Participants with moderate (*p* = 0.035) or high (*p* = 0.008) levels of social anxiety decreased their CU more than those in the control group did (*p* = 0.021). In addition, Walukevich-Dienst et al. [39] compared an online PFI focusing on cannabis problems to personalized normative feedback and found no difference between the groups in terms of CU or consequences at the one-month follow-up. However, women in the intervention group reported fewer CU problems than those in the control group did (*p* = 0.012).

In contrast to these results, Riggs et al. [38] reported that an online PFI focusing on behavioral strategies had significantly better results in terms of CU frequency compared to healthy stress management six weeks post-treatment. In addition, among the participants in the intervention group, women used more protective behavioral strategies than men did (*p* < 0.05).

Moreover, three other online interventions were administered to participants between the ages of 18 and 25 with no restrictions relating to educational status. First, Mason, Zaharakis, Russell et al. [41] evaluated the efficacy of a peer network counseling text (PNC-txt) intervention based on MI for 30 days compared to that of a waiting list. At three months, the participants in the intervention group reported significantly fewer cannabis problems (*p* = 0.04) and negative urine results (*p* = 0.03), but not a lower CU frequency, quantity, craving, or peer network health compared to those of the control group. CUD severity moderated the relationship between treatment and cannabis problems, such that participants with moderate or high CUD severity had significantly reduced CU consequences compared to those with low CUD severity. No difference was found between groups for CU frequency or consequences, urine drug screen, peer relationships, or craving. Moreover, among the participants with less severe CUD, those in the PNC-txt group reported lower frequencies of CU, craving, and interpersonal problems than those in the control group did. These results contrast with the authors’ previous study [41], in which a moderate or high CUD severity moderated the relationship between the treatment and CU consequences. Additionally, Bonar et al. [42] evaluated a social media intervention in emerging adults based on MI and CBT compared to content unrelated to substance use and mental health. The only difference between the groups was the total days in which the participants vaped cannabis (*p* = 0.02).

Finally, Walton et al. [34] evaluated a single intervention based on MI delivered via a computer or a therapist compared to that delivered via a brochure. The participants in the computer-based intervention reported fewer cannabis consequences at the three-month follow-up (*p* < 0.05) compared to those of the brochure group, but not at the 6- or 12-month follow-ups. They also reported using fewer other substances than the brochure group did at the three- and six-month follow-ups (*p* < 0.01). However, at the 12 month follow-up, all three groups did not differ in relation to any outcomes.

## 4. Discussion

The aim of this review was to examine all the psychological interventions specifically targeting CU among adolescents and young adults. To aid in our discussion, we establish the following definitions: interventions consisting of two or fewer sessions are considered to be brief; those with between three and ten sessions are classified as long, and all other interventions are deemed to be intensive. However, we made an exception for Mason et al. [40,41], whose interventions were regarded as brief because the total estimated time taken to complete the intervention was approximately 20 min. We also considered that any intervention that was not delivered in-person to be an online study.

To target the impact of specific intervention techniques on CU, we have designed a constrained set of inclusion criteria. Our inclusion criteria are limited to RCTs involving adolescents and young adults with CU exclusively and have been designed in line with the inclusion criteria used by Gates et al. [44], who conducted a systematic review focusing on CU among adults. We did not include participants with substance use disorders other than the cannabis one as the profile of participants would be different, which means the framework and goals of the interventions would be too. As a result, only 25 RCTs of psychological interventions exclusively targeting CU among youths up to 25 years old met the inclusion criteria. Out of the 25 RCT studies included in our review, 23 out of 25 included MI. 17 studies were in-person, and nine were online. We note that for the comparative study conducted by Walton et al. [34], we considered each of their in-person and online interventions individually for the purpose of this discussion. Finally, 17 interventions were brief, four were long, and four were intensive.

No recent systematic review or meta-analysis that we are aware of has replicated our set of inclusion criteria for CU among adolescents and young adults. For example, Halladay et al. [13] included non-RCT studies in their meta-analysis and focused on brief interventions with youth who infrequently engaged in CU. Only 13 out of the 25 studies included in our systematic review were also included in theirs, while the remaining 12 studies in our review were either more recent or did not meet their brief intervention criteria. Moreover, a systematic review by Beneria et al. [15] included online interventions that targeted all substance use, rather than solely CU. Out of the 25 studies included in our review, only eight were also included in theirs. Finally, Steele and al. [45] included interventions targeting adolescents with all substance use disorders, without targeting CUD specifically.

Out of the 25 studies included in our review, 14 had a significant outcome related to CU. In the following discussion, we independently analyze the impact of three key variables on the intervention outcomes: the length of the intervention, the delivery mode, and whether it included specific techniques targeting adolescent behavior.

Out of the 25 studies included in our review, 17 were brief interventions, four were long interventions, and four were intensive interventions. Of the 17 brief interventions, 11 (65%) showed significant results at the follow-up, and of the four long interventions, three (75%) showed significant results at the follow-up. None of the four intensive interventions, regardless of the techniques used, had significant results at the follow-up. Consistent with Steel et al. [45], we found that intensive interventions did not reduce the frequency of CU [23,24,25,43]. They also had high dropout rates ranging from 33% [24] to 57% [25,43]. Furthermore, we did not find any significant differences in the results between the short and long interventions included in our review. Two reviews on adolescents demonstrated the effectiveness of brief interventions on the CU outcomes [13,45]. However, another review on psychosocial interventions among adults with CUD concluded that interventions longer than four sessions had better results than the brief interventions did [44].

Of the nine online interventions included, eight (89%) showed significant results at the follow-up, demonstrating the effectiveness of online interventions for adolescents and young adults. This contradicts findings from a meta-analysis on online interventions for CU [15] that concluded that these were not effective in reducing CU among youths. Nevertheless, the authors of this study acknowledged in their conclusions, that online interventions focused solely on CU did show promising results. This point underscores the crucial role of inclusion criteria in accurately assessing the effectiveness of an intervention. In our review, we specifically focus on interventions for CU among youths, while the meta-analysis we compared our findings to those in [15] included interventions that targeted substance use in general. By narrowing our focus and carefully selecting our inclusion criteria, we were able to better highlight the effectiveness of online interventions for CU among youths.

It is worthwhile noting that online interventions have been proven to be also effective in very specific cases such as for youths with less severe CUD symptoms [40], for youths with social anxiety [35], or for youths with a family history of substance use [37]. Furthermore, brief online interventions appeared to be more effective for women when it came to CUD symptoms [36], CU consequences [39], or protective strategies [38]. This could support the importance of tailoring intervention according to gender, the severity of CUD, and family and personal psychiatry histories.

Out of the ten interventions that relied on specific strategies tailored for adolescents, eight (80%) yielded significant results at follow-up. In contrast, only six out of the 15 interventions (40%) that did not incorporate specific strategies demonstrated significant results. Interventions with specific techniques aimed at improving the engagement and/or targeting developmental characteristics of adolescents were found to be more effective for reducing CU or its consequences. To improve engagement, three of these interventions relied on daily text messages [27,40,41], while another used the presence of an e-coach. Moreover, to target developmental characteristics of adolescents, some of these intervention focused on emotion regulation and family or peer relationships [26,33,35,40,41]. These interventions relied on communication skills, problem solving, coping skills, and alternative activities. Our conclusions regarding the use of specific techniques are consistent with those of a systematic review supporting the importance of interventions focusing on peer relationships in the field of mental health among adolescents [46].

Finally, it is worthwhile noting that the combination of MET, CBT, and CM appeared to be effective during the treatment, but this result was not maintained at the follow-up [22,23,24]. Furthermore, integrating optional CBT sessions into an intervention based on MI has shown promising results for reducing CU [19,22,33,42]. In fact, a higher attendance at CBT optional sessions was correlated with a decrease in CU frequency and consequences [29,30]. Although our review shows promising results regarding the effectiveness of the combination of MI and CBT in reducing CU, more targeted research may be needed to confirm this.

It is important to note that the studies included in our review had some methodological weaknesses as they presented a high ROB in several domains. For example, only six of the 25 interventions assessed a diagnostic of CUD. Furthermore, a limited number of studies assessed psychiatric disorders and other substance use; although, CU is a risk factor for developing psychiatric disorders, particularly during adolescence [4,7,9]. Thus, it would be necessary for future studies to distinguish between youths who engage in CU and those with CUD, as well as to assess psychiatric disorders, including other substance use. In addition, a large number of studies presented a high ROB in regard to participant blinding, personnel and outcome assessments, as well as attrition bias. Although, these biases are common in psychological trials, future trials could add an objective assessment, blind the outcome assessors, and perform appropriate sensitivity analyses of missing data.

It is also important to note the limitations of our review. Firstly, only three databases were searched, and thus, it might be possible that some studies were not retrieved automatically using keywords. Secondly, the study’s scope was limited by the inherent constraints of conducting a systematic review of the literature. While the review enabled us to consolidate the research conducted on the subject, conducting a meta-analysis in the future could yield a more accurate and scientific quantification of our conclusions, thus leading to a more precise understanding of the topic.

## 5. Conclusions

Despite the limitations stated above, our review highlights promising results regarding the efficacy of certain interventions in reducing CU among adolescents. These interventions include non-intensive interventions, online interventions, and interventions targeting social and emotional skills. In conclusion, more targeted research around these techniques may be needed in order to better understand the most effective interventions for treating CU among youths.

## Figures and Tables

**Table 1:** Characteristics of interventions.

	*n*	Mean Age (SD)	Gender (Female %)	Follow-Up	Length of Treatment	Mode	Treatment Group	Control Group
Martin & Copeland (2008) [19]	40	16.5 (1.3)	33	3 months post-intervention	2 sessions	In person	MET and CBT	Delayed treatment control
De Gee et al. (2014) [20]	119	18.1 (1.8)	26	3 months post-intervention	2 sessions (60–90 min)	In person	MI	1 information session (56 min)
McCambridge et al. (2008) [21]	326	17.95 (NR)	31	3 and 6 months post-intervention	1 session	In person	MI	Drug information and giving advice
Carroll et al. (2006) [22]	136	21 (2.1)	11	3 and 6 months post-intervention	8 sessions	In person	MET, CBT, and CM	(1) MET and CBT (2) Drug counseling with CM (3) Drug counseling without CM
Stanger et al. (2009) [23]	69	16 (NR)	17	3, 6, and 9 months post-treatment	14 sessions (40 min): 14 sessions with the adolescent and 14 sessions with the parents	In person	MET, CBT, CM (abstinence), and family management	MET, CBT, CM (attendance), and parent psychoeducation
Stanger et al. (2015) [24]	153	15.8 (1.3)	11	3, 6, 9, and 12 months post-intervention	14 sessions (40 min): 14 sessions with the adolescent and 14 sessions with the parents	In person, computerized	MET, CBT, CM, and parent training	(1) MET, CBT, CM (2) MET/CBT
Stanger et al. (2020) [25]	59	16.4 (1.8)	71	Post-intervention	25 sessions	In person	Phase 1: CM and WMT Phase 2: standard CM and WMT or enhanced CM + WMT	Phase 1: CM Phase 2: Standard CM, or enhanced CM
Spirito et al. (2018) [26]	69	15.8 (NR)	39	3 and 6 months post-baseline	1 session (90 min): 1 session with the adolescent and 1 session with the parents	In person	MET and family checkup	1 session of psychoeducation (60 min): 1 session with the adolescent and 1 session with the parents separately
Shrier et al. (2018) [27]	70	20.7 6 (NR)	61	3 month post-intervention	2 sessions	In person	MET, mobile self-monitoring, and feedback messages	(1) Met and mobile self-monitoring (2) MET only
Walker et al. (2006) [28]	97	15.75 (1.32)	52	3 months post-intervention	2 sessions	In person	MET	Delayed treatment control
Walker et al. (2011) [29]	310	15.97 (1.24)	39	3 and 12 months post- baseline	2 sessions (45–50 min)	In person	MET and optional sessions of CBT	(1) Educational feedback control + 4 optional sessions of individual CBT (2) Delayed feedback control
Walker et al. (2016) [30]	252	15.84 (0.96)	32	6, 9, 12, and 15 months post-baseline	5 sessions	In person	MET and optional sessions of CBT	2 sessions of MET and optional sessions of individual CBT
Lee et al. (2013) [31]	212	20.0 (1.6)	45	3 and 6 months post-baseline	1 session (60 min)	In person	MI	Assessment only
Bernstein et al. (2009) [32]	210	NR (14–21)	66	3 and 12 months post-baseline	1 session (20–30 min)	In person	MI	(1) Assessed control (2) Non-assessed control
Prince et al. (2020) [33]	37	20.36 (1.71)	35	1, 3, and 6 months post-intervention	4 sessions	In person	MET, CBT, and exercise condition	MET and CBT
Walton et al. (2013) [34]	328	16.3 (1.6)	67	1, 3, and 12 months post-intervention	1 session	Online In person	MI delivered by computer or therapist	Brochure
Buckner et al. (2020) [35]	63	19.1 (NR)	83	2 weeks post-baseline	1 session	Online	PFI for negative affect and cannabis based on MI	Assessment only
Elliott et al. (2014) [36]	317	19.34 (1.22)	52	1 month post-intervention	1 session (45 min)	Online	Educational program and PFI	Assessment only
Lee et al. (2010) [37]	341	18.03 (0.31)	55	3 and 6 months post-baseline	1 session	Online	PFI	Assessment only
Riggs et al. (2018) [38]	298	19.97 (2.0)	49	6 weeks post-intervention	1 session	Online	PFI based on MI	Healthy stress management
Walukevich-Dienst et al. (2019) [39]	227	19.83 (1.43)	77	1 month post-baseline	1 session	Online	PFI based on MI	Personalized normative feedback
Mason, Zaharakis, Moore et al. (2018a) [40]	101	20.33 (1.76)	43	1, 2, and 3 months post-baseline	30 days	Text	Peer network counseling based on MI	Assessment only
Mason, Zaharakis, Russell et al. (2018b) [41]	30	20.75 (NR)	50	1, 2, and 3 months post-baseline	30 days	Text	Peer network counseling text based on MI	Delayed treatment control
Bonar et al. (2022) [42]	149	21.0 (2.2)	56	3 and 6 months post-baseline	8 sessions	Online e-coach	MI and CBT	Manuel-based content unrelated to substance use and mental health
Sweeney et al. (2018) [43]	87	16.2 (1.6)	16	3 and 6 months post-intervention	25 sessions (30 min)	In person	Cognitive training involved an adaptive procedure	Cognitive training involved a static procedure

CBT: cognitive and behavioral therapy; CM: contingence management; FU: follow-up; MET: motivational enhancement therapy; MI: motivational interviewing; PFI: personalized feedback intervention.

**Table 2:** Study characteristics and key findings.

	Inclusion	Exclusion	Measures	Key Findings (Last Follow-Up)	Dropouts (%)
Martin & Copeland (2008) [19]	(a) 14–19 years old (b) CU ≥ once in the last month	(a) Significant cognitive impairment (b) Use of 80 g of alcohol per day and/or other illicit substances more than twice weekly in the past 90 days (c) Treatment for substance use in the past 90 days	CU frequency and quantity: TLFB (90 days): Number of CUD symptoms: GAIN	Significant for frequency (*p* = 0.032) and quantity (*p* = 0.021) of CU, and number of CUD symptoms (*p* = 0.04)	20
De Gee et al. (2014) [20]	(a) 14–21. years (b) CU ≥ once per week (c) No intention to seek help	(a) Cognitive impairment (b) Treatment for substance use disorder in the past two months (c) Use of illicit drugs more than twice weekly during the past 3 months	CU frequency and quantity: CUPIT CUD Severity: CUPIT, SDS Psychosocial functioning: YSR	Not significant	17.65
McCambridge et al. (2008) [21]	(a) 16–19 years old (b) CU ≥ once per week	NR	CU frequency and quantity: Self-reporting Severity of CUD: SDS CU consequences: CPQ Alcohol use: AUDIT Nicotine use: Fagerstrom test	Not significant	19.02
Carroll et al. (2006) [22]	(a) 18–25 years old (b) Referred by the office of adult probation (c) Presence of CUD (SCID)	(a) Severe substance use disorder requiring treatment (b) Absence for current CUD or failure to submit a positive urine drug screen at baseline (c) Physical dependence on alcohol or opioids (d) Psychotic disorder (e) Treatment for CUD in the past 60 days (f) Current homicidal risk (g) Score < 25 on the mini-mental state examination (h) Severe medical problems	CU frequency: TLFB, urine and breath analyses Psychosocial functioning: ASI	Significant for CU: MET and CBT vs. DC (*p* = 0.02) Significant for treatment retention for CM vs. no CM (d = 0.42, 95% CI = 0.05, 0.84) and MET, CBT, and CM (d = 0.47, 95% CI = 0.12, 0.81)	20.59
Stanger et al. (2009) [23]	(a) 12–18 years old (b) CU during the past 30 days or a positive urine drug screen (c) Living with a parent/guardian who agreed to participate	(a) Active psychosis (b) Current suicidal behavior (c) Severe medical illness (VISDI) (d) Alcohol, opiate, or cocaine dependence requiring treatment	CU: VSDI, TLFB, urine drug screen Psychopathology: VSDI, CBCL/YSR Parenting measures: APQ	Not significant	40.58
Stanger et al. (2015) [24]	(a) 12–18 years old (b) CU during the last 30 days or a positive urine drug screen (c) CUD (VSDI) (d) living with a parent or guardian willing to participate	(a) Substance use disorder other than cannabis (b) cognitive impairment	CU: VSDI, TLFB, urine drug screen Psychopathology: CBCL Parenting measures: APQ	Not significant	33.33
Stanger et al. (2020) [25]	(a) 12–26 years old (b) CU during the last 30 days or a positive urine drug screen (c) Presence of CUD (DSM-5)	(a) Active psychosis (b) Severe medical or psychiatric condition limiting participation (DSM5) (c) Substance use disorders other than CUD (d) Pregnant or breastfeeding	CU frequency: TLFB (90 days) Visual spatial working memory: computerized task urine drug screen	Not significant	57
Spirito et al. (2018) [26]	(a) 13–18 years old (b) Living with a parent or a legal guardian (c) CU ≥ 3 times in the last 90 days (d) History of school truancy in the past school year	Psychiatric or development disorders preventing participation	CU frequency and quantity: TLFB (90 days) Frequency and quantity of alcohol use: ADQ Other substance use: urine drug screen Parent-teen interaction: FAsTask	Significant for CU frequency (d = 0.49, 95% CI = 0.13, 0.84), parental monitoring (d = 0.58, 95% CI = −1.09, −0.05), parent (d = 0.42, 95% CI = −0.94, 0.11) and adolescent problem solving (d = −0.66, 95% CI = −1.17, −0.11)	13.04
Shrier et al. (2018) [27]	(a) 15–24. years (b) CU ≥ 3 times per week	(a) Medically or emotionally unstable (ADI) (b) Intoxicated (c) Reported heavy or dangerous use of substances other than cannabis in past 30 days (d) Parenting youth	CU frequency: TLFB (30 days), momentary reports Consequences of cannabis: POSIT CU craving: momentary reports	Significant for momentary cannabis desire (MOMENT vs. MET only; *p* = 0.006) and for momentary CU after a trigger (MOMENT vs. No-message; *p* = 0.02)	37.14
Walker et al. (2006) [28]	(a) 14–19 years old (b) Grade 9–12 (c) CU ≥ 9 times in the past month	Thought disorder	Frequency of cannabis and other substances: GAIN (60 days) Number of CUD symptoms: GAIN	Not significant	5
Walker et al. (2011) [29]	(a) 14–19 years old (b) Grade 9–12 (c) CU ≥ 9 days in the past 30 days	Thought disorder	Frequency of cannabis and other substances: GAIN-I (60 days) Number of CUD symptoms: GAIN-I CU consequences: MPI Other treatment: GAIN-I	Not significant	9
Walker et al. (2016) [30]	(a) 14–19 years old (b) Grade 9–12 (c) CU ≥ 9 days in the past 30 days	Severe medical or psychiatric condition	Frequency of cannabis and other substances: GAIN-I (60 days) Number of CUD symptoms: GAIN-I CU consequences: MPI	Not significant	9.13
Lee et al. (2013) [31]	(a) College students (b) 18–25 years old (c) CU ≥ 5 times in the past month	NR	CU frequency: TLFB (30 days) CU quantity: DDQ (60 days) CU consequences: RMPI	Not significant	17.45
Bernstein et al. (2009) [32]	(a) 14–21 years old (b) CU ≥ 3 times in the past 30 days or reported risky behaviors associated with CU	(a) Absence of AUD (b) Treatment for substance use disorder (c) In custody or institutionalized (d) Presented for a rape exam (e) Psychiatric evaluation for suicide precaution	CU frequency: TLFB (30 days) CU consequences: AIC	Significant for CU frequency among participants who reported CU in the last 30 days (*p* < 0.027)	29.04
Prince et al. (2020) [33]	(a) 18–25 years old (b) At least fifth grade (c) CU ≥ least 3 times per week	(a) Treatment for substance use disorder or psychiatric problems (b) Substance use disorder (drug abuse screening test-10) (c) Absence of criminal justice involvement	CU frequency and quantity: EMA Protective behavioral strategies: EMA Alcohol use: DDQ	Significant for CU	NR
Walton et al. (2013) [34]	(a) 12–18 years old (b) CU during the past year	NR	CU frequency and other substances: add health items (3 months) CU consequences: RAPI, SDS Alcohol use: AUDIT	Not significant	16.2
Buckner et al. (2020) [35]	(a) Undergraduate student at Louisiana State University (psychology pool) (b) ≥18 years old (c) CU in the last month	NR	CU frequency and quantity: TLFB (2 weeks) Social anxiety: SIAS-S Positive and negative affect: PANSA	Significant for frequency of CU only for moderate (*p* = 0.035) or high levels of social anxiety (*p* = 0.008)	38
Elliott et al. (2014) [36]	(a) Students from psychology courses (b) CU ≥ once in the last month	NR	CU frequency: self-reporting (past month) Number of CUD symptoms: AUDADIS-IV CU Consequences: RMPI	Not significant	1.58
Lee et al. (2010) [37]	(a) College students (b) 17 to 19 (c) CU during the past 3 months	NR	CU: GAIN-I CU consequences: RMPI	Not significant	5.57
Riggs et al. (2018) [38]	(a) University student (b) Recreational CU (c) CU ≥ twice per week	NR	CU frequency: self-reporting CU consequences: self-reporting Strategies: PBSM	Significant for hours (*p* < 0.05), days (*p* < 0.01), and periods of CU (*p* < 0.05) per week and weeks per month (*p* < 0.01)	23.59
Walukevich-Dienst et al. (2019) [39]	(a) Undergraduate student at Louisiana State University (b) Past month CU (c) ≥1 CU problem during the last three months	<18 years old	CU frequency: MUF CU consequences: MPS	Significant for CU consequences only for women (*p* < 0.01)	22.03
Mason, Zaharakis, Moore et al. (2018b) [40]	(a) 18–25 years old (b) CU ≥ 3 times per week (c) Presence of CUD (CUDIT) CUDIT-R ≥ 8	Treatment for substance use disorder in the last 3 months	CU frequency: CUDIT-R, ASSIST (30 days), urine drug screen CU consequences: YBSR, MPI Peer network health: YASNA	Significant for frequency of heavy CU (*p* = 0.005) and interpersonal problems (*p* = 0.011)	4.95
Mason, Zaharakis, Russell et al. (2018a) [41]	(a) 18–25 years old (b) Presence of CUD (CUDIT) (c) Positive urine drug screen (d) Absence of alcohol use disorder	Treatment for substance use disorder in the last 90 days	CU frequency and quantity: TLFB (30 days), urine drug screen CU consequences: MPI Craving: EMA Peer network health: YASNA	Significant for CU consequences (*p* = 0.04), and urine drug screen (*p* = 0.03)	13.3
Bonar et al. (2022) [42]	(a) 18–25 years old (b) CU ≥ 3 times per week in the past month	NR	CU frequency and alcohol use: TLFB (30 days)	Significant only for frequency of vaped cannabis (*p* = 0.02)	10.74
Sweeney et al. (2018) [43]	(a) 14–21 years old (b) Undergoing a treatment for CUD	(a) Untreated Axis I psychiatric disorders (b) Use ≥ 4 times per week of any substance other than caffeine, nicotine, or cannabis (c) Cognitive impairment	Frequency and quantity of CU, alcohol, and tobacco: TLFB (30 days) Psychosocial functioning: GAIN-I, DERS Therapeutic alliance: WAI-SR urine drug screen	Not significant	57.47

ADI: adolescent diagnostic interview; AIC: Adolescent Injury Checklist; APQ: Alabama Parenting Questionnaire; ASI: Addiction Severity Index; ASSIST: Alcohol, Smoking, and Substance Involvement Screening Test; AUDADIS-IV: Alcohol Use Disorder and Associated Disabilities Interview Schedule—IV; AUDIT: Alcohol Use Disorder Identification Test; CBC: Child Behavior Checklist; CBT: cognitive and behavioral therapy; CM: contingence management; CU: cannabis use; CUDIT: Cannabis Use Disorder Identification Test; CUDIT-R: Cannabis Use Disorder Identification Test—Revised; DC: drug counseling; DDQ: Daily Drinking Questionnaire; DERS: Difficulties in Emotion Regulation Scale; DSM5: Diagnostic and Statistical Manual of Mental Disorders; EMA: Ecological Momentary Assessment; FAsTask: Videotaped Family Assessment Task; GAIN: Global Appraisal of Individual Needs; MET: motivational enhancement therapy; MPI: Marijuana Problems Inventory; MPS: Marijuana Problems Scale; MUF: Marijuana Use Form; NR: not reported; PANSA: Positive Affect and Negative Affect Schedule; PBSM: Protective Behavioral Strategies for Marijuana; PFI: personalized feedback intervention; RAPI: Rutgers Alcohol Problems Index; SIAS-S: Social Interaction Anxiety Scale; VSDI: Vermont Structured Diagnostic Interview; TLFB: Timeline Follow Back; WAI-SR: Working Alliance Inventory Short Revised; WMT: working memory training; YASNA: Young Adult Social Network Assessment; YBSR: Youth Risk Behavior Survey; YSR: Youth Self-Reporting.

## Data Availability

The data presented in this study are available on request from the corresponding author.

## References

[1] Substance Abuse and Mental Health Services Administration (2020). Key Substance Use and Mental Health Indicators in the United States: Results from the 2020 National Survey on Drug Use and Health.

[2] (2013). American Psychiatric Association Diagnostic and Statistical Manual of Mental Disorders.

[3] European Monitoring Centre for Drugs and Drug Addiction (2022). European Drug Report: Trends and Developments.

[4] Blest-Hopley G., Colizzi M., Giampietro V., Bhattacharyya S. (2020). Is the Adolescent Brain at Greater Vulnerability to the Effects of Cannabis? A Narrative Review of the Evidence. Front. Psychiatry.

[5] Hasbi A., Madras B.K., George S.R. (2023). Endocannabinoid System and Exogenous Cannabinoids in Depression and Anxiety: A Review. Brain Sci..

[6] Hammond C., Allick A., Park G., Rizwan B., Kim K., Lebo R., Nanavati J., Parvaz M., Ivanov I. (2022). A Meta-Analysis of fMRI Studies of Youth Cannabis Use: Alterations in Executive Control, Social Cognition/Emotion Processing, and Reward Processing in Cannabis Using Youth. Brain Sci..

[7] Gobbi G., Atkin T., Zytynski T., Wang S., Askari S., Boruff J., Ware M., Marmorstein N., Cipriani A., Dendukuri N. (2019). Association of Cannabis Use in Adolescence and Risk of Depression, Anxiety, and Suicidality in Young Adulthood: A Systematic Review and Meta-analysis. JAMA Psychiatry.

[8] Albaugh M.D., Ottino-Gonzalez J., Sidwell A., Lepage C., Juliano A., Owens M.M., Chaarani B., Spechler P., Fontaine N., Rioux P. (2021). Association of Cannabis Use During Adolescence With Neurodevelopment. JAMA Psychiatry.

[9] Owens M.M., Albaugh M.D., Allgaier N., Yuan D., Robert G., Cupertino R.B., Spechler P.A., Juliano A., Hahn S., Banaschewski T. (2022). Bayesian causal network modeling suggests adolescent cannabis use accelerates prefrontal cortical thinning. Transl. Psychiatry.

[10] Hines L.A., Freeman T.P., Gage S.H., Zammit S., Hickman M., Cannon M., Munafo M., MacLeod J., Heron J. (2020). Association of High-Potency Cannabis Use With Mental Health and Substance Use in Adolescence. JAMA Psychiatry.

[11] Mericle A.A., Arria A.M., Meyers K., Cacciola J., Winters K.C., Kirby K. (2015). National Trends in Adolescent Substance Use Disorders and Treatment Availability: 2003–2010. J. Child Adolesc. Subst. Abus..

[12] Dennis M., Godley S.H., Diamond G., Tims F.M., Babor T., Donaldson J., Liddle H., Titus J.C., Kaminer Y., Webb C. (2004). The Cannabis Youth Treatment (CYT) Study: Main findings from two randomized trials. J. Subst. Abuse Treat..

[13] Halladay J., Scherer J., MacKillop J., Woock R., Petker T., Linton V., Munn C. (2019). Brief interventions for cannabis use in emerging adults: A systematic review, meta-analysis, and evidence map. Drug Alcohol Depend..

[14] Li L.Y., Mann R.E., Wickens C.M. (2019). Brief Interventions for Cannabis Problems in the Postsecondary Setting: A Systematic Review. Int. J. Ment. Health Addict..

[15] Beneria A., Santesteban-Echarri O., Daigre C., Tremain H., Ramos-Quiroga J.A., McGorry P.D., Alvarez-Jimenez M. (2022). Online interventions for cannabis use among adolescents and young adults: Systematic review and meta-analysis. Early Interv. Psychiatry.

[16] Olmos A., Tirado-Muñoz J., Farré M., Torrens M. (2018). The efficacy of computerized interventions to reduce cannabis use: A systematic review and meta-analysis. Addict. Behav..

[17] Boumparis N., Loheide-Niesmann L., Blankers M., Ebert D.D., Korf D., Schaub M.P., Spijkerman R., Tait R.J., Riper H. (2019). Short- and long-term effects of digital prevention and treatment interventions for cannabis use reduction: A systematic review and meta-analysis. Drug Alcohol Depend..

[18] Cochrane Handbook for Systematic Reviews of Interventions. https://training.cochrane.org/handbook/current.

[19] Martin G., Copeland J. (2008). The adolescent cannabis check-up: Randomized trial of a brief intervention for young cannabis users. J. Subst. Abuse Treat..

[20] de Gee E.A., Verdurmen J.E.E., Bransen E., de Jonge J.M., Schippers G.M. (2014). A randomized controlled trial of a brief motivational enhancement for non-treatment-seeking adolescent cannabis users. J. Subst. Abuse Treat..

[21] McCambridge J., Slym R.L., Strang J. (2008). Randomized controlled trial of motivational interviewing compared with drug information and advice for early intervention among young cannabis users. Addiction.

[22] Carroll K.M., Easton C.J., Nich C., Hunkele K.A., Neavins T.M., Sinha R., Ford H.L., Vitolo S.A., Doebrick C.A., Rounsaville B.J. (2006). The use of contingency management and motivational/skills-building therapy to treat young adults with marijuana dependence. J. Consult. Clin. Psychol..

[23] Stanger C., Budney A.J., Kamon J.L., Thostensen J. (2009). A randomized trial of contingency management for adolescent marijuana abuse and dependence. Drug Alcohol Depend..

[24] Stanger C., Ryan S.R., Scherer E.A., Norton G.E., Budney A.J. (2015). Clinic- and Home-Based Contingency Management Plus Parent Training for Adolescent Cannabis Use Disorders. J. Am. Acad. Child Adolesc. Psychiatry.

[25] Stanger C., Scherer E.A., Vo H.T., Babbin S.F., Knapp A.A., McKay J.R., Budney A.J. (2020). Working memory training and high magnitude incentives for youth cannabis use: A SMART pilot trial. Psychol. Addict. Behav..

[26] Spirito A., Hernandez L., Cancilliere M.K., Graves H.R., Rodriguez A.M., Operario D., Jones R., Barnett N.P. (2018). Parent and Adolescent Motivational Enhancement Intervention for Substance-Using, Truant Adolescents: A Pilot Randomized Trial. J. Clin. Child Adolesc. Psychol..

[27] Shrier L.A., Burke P.J., Kells M., Scherer E.A., Sarda V., Jonestrask C., Xuan Z., Harris S.K. (2018). Pilot randomized trial of MOMENT, a motivational counseling-plus-ecological momentary intervention to reduce marijuana use in youth. mHealth.

[28] Walker D.D., Roffman R.A., Stephens R.S., Wakana K., Berghuis J. (2006). Motivational enhancement therapy for adolescent marijuana users: A preliminary randomized controlled trial. J. Consult. Clin. Psychol..

[29] Walker D.D., Stephens R., Roffman R., DeMarce J., Lozano B., Towe S., Berg B. (2011). Randomized controlled trial of motivational enhancement therapy with nontreatment-seeking adolescent cannabis users: A further test of the teen marijuana check-up. Psychol. Addict. Behav..

[30] Walker D.D., Stephens R.S., Blevins C.E., Banes K.E., Matthews L., Roffman R.A. (2016). Augmenting brief interventions for adolescent marijuana users: The impact of motivational check-ins. J. Consult. Clin. Psychol..

[31] Lee C.M., Kilmer J.R., Neighbors C., Atkins D.C., Zheng C., Walker D.D., Larimer M.E. (2013). Indicated prevention for college student marijuana use: A randomized controlled trial. J. Consult. Clin. Psychol..

[32] Bernstein E., Edwards E., Dorfman D., Heeren T., Bliss C., Bernstein J. (2009). Screening and Brief Intervention to Reduce Marijuana Use Among Youth and Young Adults in a Pediatric Emergency Department. Acad. Emerg. Med..

[33] Prince M.A., Collins R.L., Wilson S.D., Vincent P.C. (2020). A preliminary test of a brief intervention to lessen young adults’ cannabis use: Episode-level smartphone data highlights the role of protective behavioral strategies and exercise. Exp. Clin. Psychopharmacol..

[34] Walton M.A., Bohnert K., Resko S., Barry K.L., Chermack S.T., Zucker R.A., Zimmerman M.A., Booth B.M., Blow F.C. (2013). Computer and therapist based brief interventions among cannabis-using adolescents presenting to primary care: One year outcomes. Drug Alcohol Depend..

[35] Buckner J.D., Zvolensky M.J., Lewis E.M. (2020). On-line personalized feedback intervention for negative affect and cannabis: A pilot randomized controlled trial. Exp. Clin. Psychopharmacol..

[36] Elliott J.C., Carey K.B., Vanable P.A. (2014). A preliminary evaluation of a web-based intervention for college marijuana use. Psychol. Addict. Behav..

[37] Lee C.M., Neighbors C., Kilmer J.R., Larimer M.E. (2010). A brief, web-based personalized feedback selective intervention for college student marijuana use: A randomized clinical trial. Psychol. Addict. Behav..

[38] Riggs N.R., Conner B.T., Parnes J.E., Prince M.A., Shillington A.M., George M.W. (2018). Marijuana eCHECKUPTO GO: Effects of a personalized feedback plus protective behavioral strategies intervention for heavy marijuana-using college students. Drug Alcohol Depend..

[39] Walukevich-Dienst K., Neighbors C., Buckner J.D. (2019). Online personalized feedback intervention for cannabis-using college students reduces cannabis-related problems among women. Addict. Behav..

[40] Mason M.J., Zaharakis N.M., Moore M., Brown A., Garcia C., Seibers A., Stephens C. (2018). Who responds best to text-delivered cannabis use disorder treatment? A randomized clinical trial with young adults. Psychol. Addict. Behav..

[41] Mason M.J., Zaharakis N.M., Russell M., Childress V. (2018). A pilot trial of text-delivered peer network counseling to treat young adults with cannabis use disorder. J. Subst. Abuse Treat..

[42] Bonar E.E., Goldstick J.E., Chapman L., Bauermeister J.A., Young S.D., McAfee J., Walton M.A. (2022). A social media intervention for cannabis use among emerging adults: Randomized controlled trial. Drug Alcohol Depend..

[43] Sweeney M.M., Rass O., DiClemente C., Schacht R.L., Vo H.T., Fishman M.J., Leoutsakos J.-M.S., Mintzer M.Z., Johnson M.W. (2018). Working Memory Training for Adolescents With Cannabis Use Disorders: A Randomized Controlled Trial. J. Child Adolesc. Subst. Abuse.

[44] Gates P.J., Sabioni P., Copeland J., Le Foll B., Gowing L. (2016). Psychosocial interventions for cannabis use disorder. Cochrane Database Syst. Rev..

[45] Steele D.W., Becker S.J., Danko K.J., Balk E.M., Saldanha I.J., Adam G.P., Bagley S.M., Friedman C., Spirito A., Scott K. (2020). Interventions for Substance Use Disorders in Adolescents: A Systematic Review.

[46] Manchanda T., Stein A., Fazel M. (2023). Investigating the Role of Friendship Interventions on the Mental Health Outcomes of Adolescents: A Scoping Review of Range and a Systematic Review of Effectiveness. Int. J. Environ. Res. Public. Health.

